# Transverse cervical vessels as recipient vessels in oral and maxillofacial microsurgical reconstruction after former operations with or without radiotherapy

**DOI:** 10.1186/s12957-015-0576-8

**Published:** 2015-05-14

**Authors:** Zhong-fei Xu, Wei-yi Duan, En-jiao Zhang, Shuang Bai, Yu Tian, Xue-xin Tan, Fa-yu Liu, Chang-fu Sun

**Affiliations:** Department of Oromaxillofacial-Head and Neck Surgery, School of Stomatology, China Medical University, No.117, Nanjing North Street, Heping District, Shenyang, Liaoning 110002 People’s Republic of China; Department of Oral Maxillofacial Surgery, School of Stomatology, China Medical University, No.117, Nanjing North Street, Heping District, Shenyang, Liaoning 110002 People’s Republic of China

**Keywords:** Transverse cervical vessel, Oral and maxillofacial, Reconstruction

## Abstract

**Background:**

The purpose of this study was to investigate the reliability and outcome of using the transverse cervical vessel (TCV) as a recipient vessel for microvascular reconstruction in patients whose vessels in the neck region are unavailable because of previous surgery or radiotherapy.

**Methods:**

Between January 2012 and August 2014, secondary head and neck reconstruction was performed using the TCV as a recipient vessel in eight patients who had undergone previous neck dissection and radiation therapy (*n* = 5). Five patients had a recurrent carcinoma, one had undergone an operation for scar release and two had been treated surgically for a second primary cancer. The anterolateral thigh flap (ALT), anteromedial thigh flap (AMT), and fibular flap were used for the reconstruction. Clinical data were recorded for each patient.

**Results:**

All of the ipsilateral transverse cervical arteries were found to be free of disease. The second free flap was revascularized using the TCVs (*n* = 6) or the external (*n* = 1) or internal (*n* = 1) jugular vein. The free flaps used for the reconstruction included the ALT flap (*n* = 6), AMT flap (*n* = 2), and fibular flap (*n* = 1). All of the flaps survived without vascular events, and the patients healed without major complications. The mean follow-up time was 11 months. One patient died of distant metastases during follow-up.

**Conclusions:**

In patients who have previously undergone neck surgery with or without radiotherapy, the TCVs are reliable and easily accessible recipient vessels for microsurgical reconstruction in the oral and maxillofacial region. If the transverse cervical vein is unavailable, the internal or external jugular vein should be dissected carefully to serve as an alternative for microvascular anastomoses.

## Background

Adjuvant radiotherapy with neck dissection for the management of head and neck cancer might have a positive effect on disease-free survival; however, it might have a negative effect on the vascular bed in the radiotherapy field [1]. In cases in which a patient develops a recurrent or second primary tumor after radiotherapy requiring reconstruction, the reconstructive surgeon faces particularly challenging problems. In such patients, local flaps are typically inadequate. Microvascular free tissue transfer is a standard reconstructive option; however, the outcome of this type of microvascular reconstruction is heavily dependent on the selection of the recipient vessel [2,3]. When the bilateral recipient vessels of first choice in the head and neck are not available because of previous surgery or irradiation, finding suitable vessels for microvascular anastomosis might be difficult [4,5]. Finding reliable recipient vessels is paramount to successful microvascular tissue transfer in patients who have undergone neck surgery, radiation therapy, or both.

The transverse cervical vessel (TCV) is occasionally described as a recipient vessel for head and neck reconstruction [6,7]. Here, we describe the technique and evaluate the reliability of using TCVs as recipient vessels in re-operative oral and maxillofacial reconstruction in cases in which other local vessels are not available or inadequate.

## Methods

### Patients

Between January 2012 and August 2014, 156 cases of microsurgical oral and maxillofacial reconstruction were performed at the Department of Oral and Maxillofacial Surgery, School of Stomatology of China Medical University. This research was approved by the ethics committee of the School of Stomatology of China Medical University. The TCVs were used for microsurgical head and neck reconstruction in eight patients who had previously undergone surgery and radiation therapy of the neck region (*n* = 5). Three patients were male, and five were female. The mean age at the time of operation was 51.8 years (range, 36 to 69 years). We performed selective neck dissections (SND, levels I to III) on the eight patients with clinically determined node-negative necks at the primary surgery. Among them, five patients underwent adjuvant radiotherapy, during which the field was confined to the operative region and did not extend to the non-dissected levels.

The prior surgeries included tumor resection, selective neck dissection (levels I to III), and flap reconstruction. An extensive description of the individual cases and the patients’ demographics is presented in Table 1.

**Table 1:** **Patient characteristics**

**Patient**	**Age/gender**	**Diagnosis**	**Prior operation**	**SND**	**Prior RT**	**First flap**	**Second flap**	**Hospitalization time**	**Complications**
1	42/F	SCC of the lower gingiva	Maxillectomy	uni	Y	None	ALT	9	None
2	36/F	ACC of FOM (recurrence)	Resection ACC FOM	bil	Y	MSAP	ALT	15	Delayed wound healing
3	59/M	ACC of the mandible (recurrence)	Marginal resection of the mandible	bil	N	Plastyma	Fibula	10	None
4	65/M	SCC of the tongue (second primary cancer)	Resection SCC of FOM	bil	Y	ALT	AMT	10	None
5	60/F	SCC of the lower gingiva (recurrence)	Hemimandibulectomy	uni	N	Fibula	ALT + AMT	19	None
6	49/F	Mouth opening limited	Maxillectomy	uni	N	None	ALT	18	Fistula and delayed wound healing
7	58/M	SCC of buccal mucosa (recurrence)	Resection SCC of buccal mucosa	uni	Y	None	ALT	9	None
8	69/F	SCC of buccal mucosa (recurrence)	Resection SCC of buccal mucosa	uni	Y	None	ALT	10	None

SND, selective neck dissection; RT, radiotherapy; SCC, squamous cell carcinoma; uni, unilateral; ALT, anterolateral thigh flap; ACC, adenoid cystic carcinoma; FOM, floor of mouth; bil, bilateral; MSAP, medial sural artery perforator flap; AMT, anteromedial thigh flap; F, female; M, male.

### Surgical procedure

All of the procedures were performed with a two-team approach. A free fibula flap, an AMT flap, and five ALT flaps were harvested on the nondominant leg to reconstruct the defects following cancer ablation. In one procedure, a chimeric flap (ALT + AMT) was harvested to reconstruct a through-to-through cheek defect (Table 1).

Technical instructions for preparation of the recipient vessels

**Figure 1:**
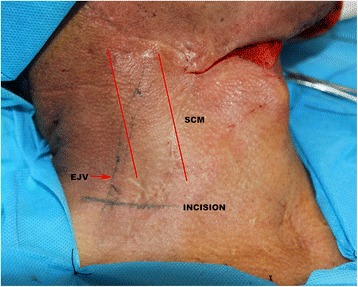
Preoperatively marked recipient vessels and designed the transverse incision parallel to the clavicle to enable exploration of TCVs. SCM, sternocleidomastoid muscle; EJV, external jugular vein.

**Figure 2:**
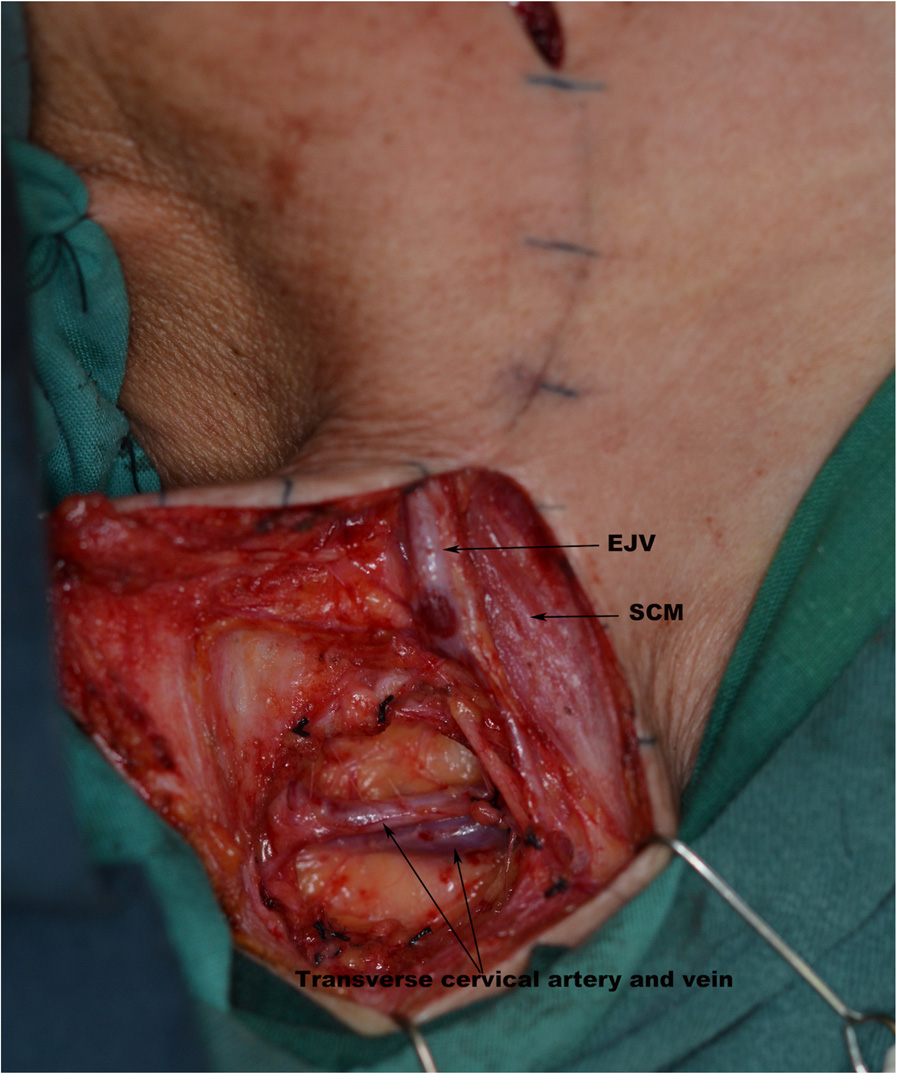
The right TCVs, located 2 cm above the clavicle and lateral to the SCM, were exposed. SCM, sternocleidomastoid muscle; EJV, external jugular vein.

The technique to expose the transverse cervical vessels has been well documented [7]. Briefly, the pulse of the transverse cervical artery (TCA) was detected preoperatively in the supraclavicular region using palpation and pencil Doppler. The defective side of the neck was selected as the surgical field. If this were the left side of the neck, the thoracic duct should not be injured. For the patients who had undergone previous surgery, the supraclavicular region (level V) was typically unexposed, and an additional incision was made 2 cm above and parallel to the clavicle. The external jugular vein should be marked and carefully dissected (Figure 1). After the sternocleidomastoid and omohyoid muscles were identified, the loose fatty tissue was carefully explored with blunt dissection in this triangle area. The TCA was easily found under the fatty tissue lateral to the sternocleidomastoid muscle. Then, the TCA was traced proximally to obtain the vessels with larger diameters. Typically, vessels at least 2- to 4-cm long could be obtained to easily facilitate microsurgery. The transverse cervical vein normally was within the fatty tissue (Figure 2). In some cases, in which the vein was small, the external jugular vein or internal jugular vein should be explored and preserved.

For reconstruction in the oral and maxillofacial region, a long pedicle of the donor flap was normally required when the TCA served as the recipient site. The flap pedicle was brought from the defect to the recipient vessels through a wide subcutaneous tunnel with a clamp. Then, microsurgery was performed. Care should be taken to position the vessels and prevent any kinks in the vessels. Arterial and venous anastomoses were typically performed with interrupted 8-0 nylon sutures and coupler.

## Results

At the time of the reoperation, the patients presented with an inaccessible or vessel-depleted neck. All of the procedures were uncomplicated; no flap was revised, and the flap failure rate was 0%. The results are schematically represented in Table 2. The time required to explore the transverse cervical vessels averaged approximately 18.3 min (12 to 24 min). The mean operating time for reconstruction was 161.3 min (133 to 195 min), and the mean flap ischemia time was 42 min (23 to 45 min). The mean pedicle length was 11.0 cm (7.5 to 15 cm), with a mean distance of 11.2 cm (8.2 to 15 cm) between the resection and the recipient vessel sites. The mean distance between the anastomosis site and the acceptor site was 8.6 cm (6.1 to 11 cm). No venous interposition grafts were needed because of the long flap pedicles. The mean hospitalization time was 12.5 days (range 9 to 19 days), and the mean postoperative follow-up was 11 months (range 1 to 23 months). One patient died after a follow-up of 18 months.

**Table 2:** **Role of the TCVs**

**Patient**	**Age/gender**	**Recipient vessels (A/V)**	**Anastomosis**	**Ischemia time (min)**	**Time of vessel dissection (min)**	**Operating time (min)**	**Distance** ^**a**^ **(cm)**	**Distance** ^**b**^ **(cm)**	**Pedicle length (cm)**
1	42/F	TCA/TCV	E-E	38	24	172	8.8	6.1	9.7
2	36/F	TCA/IJV	E-S	33	20	135	8.2	6.3	10.3
3	59/M	TCA/TCV	E-E	42	25	170	11.3	8.1	7.5
4	65/M	TCA/TCV	E-E	35	17	193	11.2	9.3	10.1
5	60/F	TCA/EJV	E-E	45	15	157	10.2	8.5	10.8
6	49/F	TCA/TCV	E-E	23	15	133	12.1	9.4	11.5
7	58/M	TCA/TCV	E-E	75	18	135	15	10	15
8	66/F	TCA/TCV	E-E	45	12	195	12.4	11	13

^a^Distance refers to bridging distance between the acceptor site and recipient vessel site, ^b^Distance refers to bridging distance between the acceptor site and anastomosis site. F, female; M, male; E-E, end to end; E-S, end to side; TCA, transverse cervical artery; TCV, transverse cervical vein; IJV, internal jugular vein; EJV, external jugular vein.

**Figure 3:**
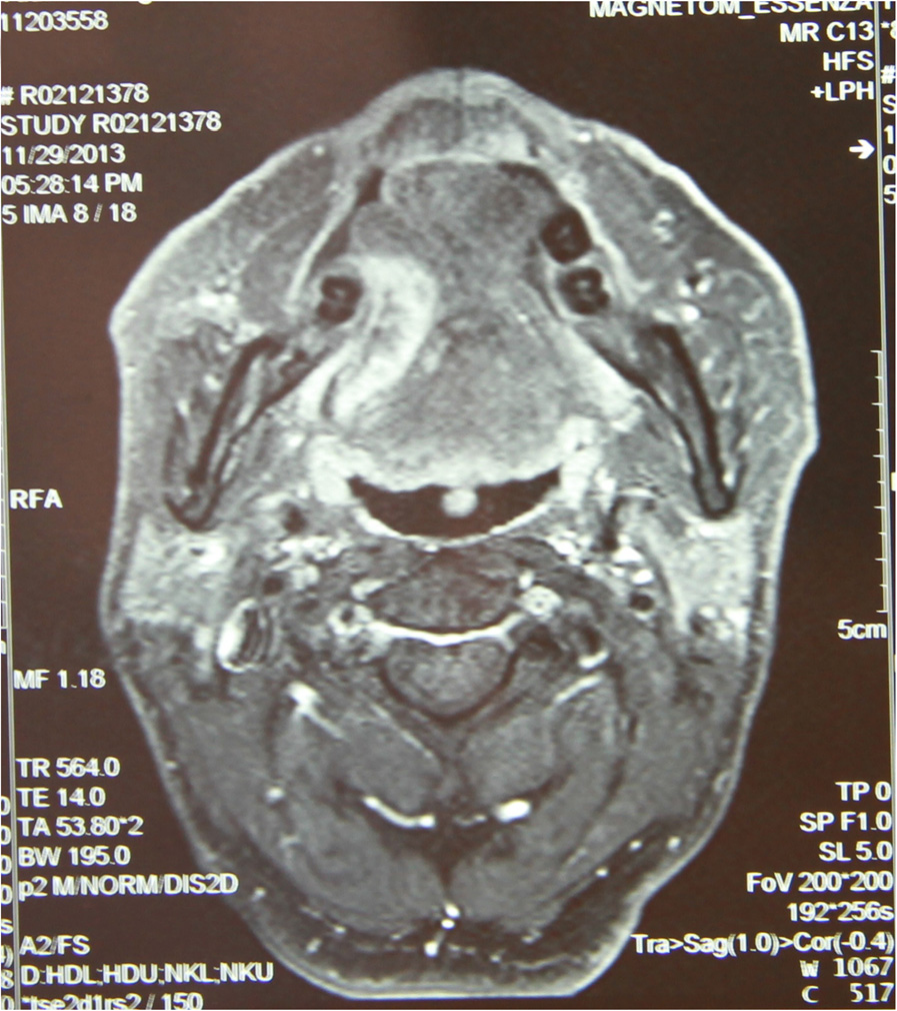
Case presentation (patient 4): a second primary carcinoma occurred in the tongue (R) 18 months after the first reconstruction with an ALT flap.

**Figure 4:**
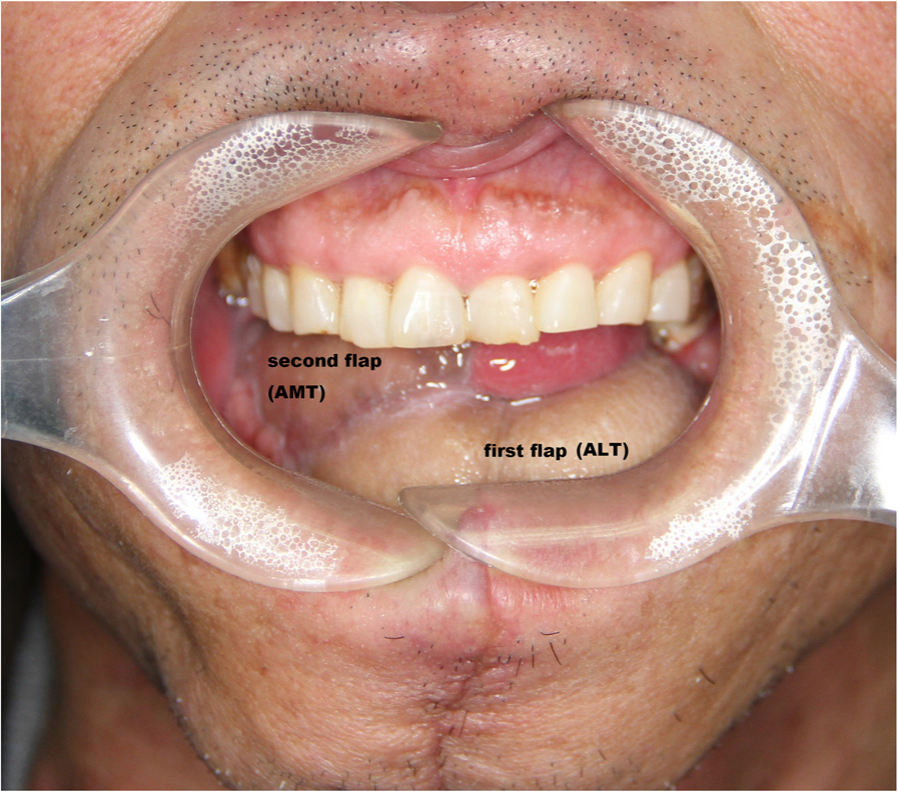
Case presentation (patient 4): the healed flap on the tongue after the 6-month follow-up. AMT, anteromedial thigh flap; ALT, anterolateral thigh flap.

A 65-year-old man presented with a primary tongue carcinoma after a marginal mandibulectomy, selective neck dissection (bil, levels I to III), and free ALT flap reconstruction for T4N0M0 gingiva cancer (patient 4 in Table 1, Figure 3). In the second reconstructive surgery, a hemiglossectomy (R) was performed, and an AMT perforator flap was used for the reconstruction of the defect. Because the bilateral neck vessels had been sacrificed during the previous neck dissection as well as severe scarring and fibrosis secondary to the postoperative radiotherapy, the right transverse cervical vessels were selected as the recipient vessels for the second reconstruction procedure. The donor site was primarily closed. The postoperative course was uneventful (Figure 4).

## Discussion

The introduction of ablative surgery followed by radiation therapy, chemotherapy, or both has led to improved local and regional disease control with better disease-free survival and a lower rate of recurrence. There is an increasing demand for salvage surgery for treatment failure or treatment-related complications, such as fistulas after adjuvant therapy, recurrence or secondary malignancies, all of which are challenging for reconstructive surgeons [6,8-10].

The rich vascular network in the head and neck include branches of the external carotid artery, the internal jugular vein, and the external jugular vein. These vessels are normally used as donor vessels for microvascular reconstruction [2,5,11]. However, in patients who have been previously treated in the neck region, the vessels might have been resected or compromised. In some patients, the vessels might not have been resected; however, dissection is difficult and unpredictable in tissues subject to fibrosis. There is some risk of damaging the surrounding tissues and inducing uncontrollable bleeding, which is one of the reasons that locating appropriate vessels for anastomosis is difficult. The surgeon must seek a safe and reliable alternative for micro-anastomosis.

Many options have been suggested for free tissue transfer in necks with few vessels suitable for surgery. These techniques include the use of internal mammary vessels [4,12], the cephalic vein [13] and thoracoacromial vascular system [14], superficial temporal vessels [15], vessels in the contralateral side of the previous neck dissection [6], a never-before-transferred free flap pedicle [16], and a wrist carrier flap [17]. Although these methods might be effective in providing a recipient site for free tissue transfer in the head and neck, they require a more invasive approach or long vein graft, which might significantly complicate the overall surgical procedure and increase the risk of failure [3,18]. In some cases, vessels in the contralateral side neck might be suitable, the TCVs should be evaluated for use in anastomosis to avoid the sacrifice of the later possibility for free flap reconstruction.

Variations in the anatomy of the transverse cervical arteries are frequently observed. The TCA originates from the thyrocervical trunk (77%); however, it could arise directly from the subclavian artery (21%) or the internal mammary artery (2%) [19]. In these cases, the preferred method is to dissect the TCVs through a separate horizon incision, as discussed above. The dissection is fast and easily reproducible, requiring no more than 20 min in most cases. In this study, the artery was consistently found to be lateral to the sternocleidomastoid muscle and above the clavicle. The arterial diameter diminishes significantly as the vessel is dissected laterally. However, the quality and pressure are typically excellent. In our experience, the transverse cervical artery is generally available and of adequate size regardless of where it originates. It is simple to obtain 2 to 3 cm from the posterior border of the sternocleidomastoid. The anastomosis site lies in the middle one third of the neck. When anastomoses to the transverse cervical vessels are performed in the supraclavicular region, the vascular pedicle typically has a straight course, which minimizes kinking of the pedicle.

The path of the transverse cervical vein is more variable. The transverse cervical vein might have a variable course as well, running deep (75%) or superficially (25%) to the omohyoid. It could drain into the external jugular vein or the subclavian vein [6]. In Yu’s report, only one vein was absent, and the diameter of the two veins was smaller than 2 mm in 33 sides [7]. In our series, one transverse cervical vein was found to be absent, and one vein was found to be inadequate. In such cases, the external or internal jugular vein served as alternative vessels, preventing the need for vein grafting. Marking the external jugular vein preoperatively and dissecting the external and internal jugular vein meticulously are therefore safe methods.

For reconstruction of the mandible or oral cavity, a subcutaneous tunnel could be created to reach the TCVs without exposing the neck, and the vascular pedicle of the flap has a straight course after it is brought from the defect to the recipient vessel. The recipient vessels have a vertical anatomical position during microsurgery and remain in this position afterwards. By this method, the risk of kinking the vessel is almost nonexistent. In this series, the distance between the anastomosis site and the acceptor site is less than 10 cm, with a mean length of 7.9 cm. Because the mainstay free flaps, such as the radial forearm flap [20], ALT [21], and AMT [22], typically having a long pedicle, they are adequate for reaching the TCVs; therefore, these flaps are preferred over flaps with short pedicles. For a fibula flap, the uses of the pedicle remain limited if a long bone is required, although the pedicle could be lengthened by using the distal portion of the fibula. In such cases, particular care should be taken. In this series, an ALT flap was used for palate reconstruction (scar release), without the need for vein grafting. The results are consistent with those found by Yu [7]. If the defect is in the middle of the face or higher, the superficial temporal vessels are a good option for the recipient site [15].

Over the previous few decades, the mainstay for neck dissection has shifted from radical surgery to a more conservative modality [23]. The work of Bajwa *et al.* [24] suggested that levels I to III selective neck dissection (SND) is effective management of the cN0 neck condition in patients with oral squamous cell carcinoma. Additionally, the survival analysis of their case series showed that SND (levels I to III) might be equivalent to SND (levels I to IV) as a staging and therapeutic procedure. In our studies, the included patients presented with clinical N0 necks before they accepted primary surgeries. Thus, we performed the levels I to III SND under those circumstances. The supraclavicular regions and posterior cervical triangles were consistently reserved to ensure that these regions were available for preparation of the TCVs for anastomosis if the patient suffered from local recurrence. For the vessel-depleted-neck cases following comprehensive neck dissections, the external jugular veins or transverse cervical veins might not be available. The stumps of the internal jugular vein, internal mammary vessels, or vein graft would be expedient options, which is the limitation of our surgical technique.

The indications for the use of the transverse cervical vessels as recipient vessels in microsurgical oral and maxillofacial reconstruction are as follows: 1. for recurrent oral and maxillofacial cancer patients who had undergone an SND (levels I to III), 2. for defects located in the lower two thirds of the oral and maxillofacial region, and 3. as a second recipient vessel, in cases in which a double free flap transfer is planned.

## Conclusion

The TCVs could be used safely as an alternative recipient site for patients whose vessels in the neck region are unavailable because of previous surgery or radiotherapy. In cases in which the transverse cervical vein is unavailable, the internal or external jugular vein should be dissected carefully and could serve as an alternative site for microvascular anastomosis.

## Consent

Written informed consent was obtained from the patient for the publication of this report and any accompanying images.
